# miR-124 and miR-506 inhibit colorectal cancer progression by targeting DNMT3B and DNMT1

**DOI:** 10.18632/oncotarget.5709

**Published:** 2015-10-15

**Authors:** Zhiheng Chen, Shaojun Liu, Li Tian, Minghao Wu, Feiyan Ai, Wuliang Tang, Lian Zhao, Juan Ding, Liyang Zhang, Anliu Tang

**Affiliations:** ^1^ Department of Pediatrics, The Third Xiangya Hospital, Central South University, Changsha, Hunan, China; ^2^ Department of Gastroenterology, The Third Xiangya Hospital, Central South University, Changsha, Hunan, China; ^3^ Hunan Key Laboratory of Nonresolving Inflammation and Cancer, Changsha, Hunan, China; ^4^ Department of Gastroenterology, The Hunan Provincial People's Hospital, Changsha, Hunan, China; ^5^ Department of Oncology, The Third Xiangya Hospital, Central South University, Changsha, Hunan, China; ^6^ Department of Neurosurgery, Xiangya Hospital, Central South University, Changsha, Hunan, China

**Keywords:** miR-124, miR-506, colorectal cancer, DNMT3B, DNMT1

## Abstract

miR-124 and miR-506 are reportedly down-regulated and associated with tumor progression in many cancers, but little is known about their intrinsic regulatory mechanisms in colorectal cancer (CRC). In this study, we found that the miR-124 and miR-506 levels were significantly lower in human CRC tissues than in controls, as indicated by qRT-PCR and *in situ* hybridization histochemistry. We also found that the overexpression of miR-124 or miR-506 inhibited tumor cell progression and increased sensitivity to chemotherapy *in vitro*. Increased miR-124 or miR-506 expression also inhibited tumor cell proliferation and invasion *in vivo*. Luciferase reporter assays and western blotting were used to determine the association between miR-124, miR-506 and their target genes, DNMTs. We further identified that miR-124 and miR-506 directly targeted DNMT3B and indirectly targeted DNMT1. The overexpression of miR-124 and miR-506 reduced global DNA methylation and restored the expression of E-cadherin, MGMT and P16. In conclusion, our data showed that miR-124 and miR-506 inhibit progression and increase sensitivity to chemotherapy by targeting DNMT3B and DNMT1 in CRC. These findings may provide novel avenues for the development of targeted therapies.

## INTRODUCTION

Colorectal cancer (CRC) is the third leading cause of cancer death worldwide, and the morbidity rate of CRC positively correlates with age [1]. The incidence of CRC continues to increase and ranks fifth among malignant tumor-related deaths in China [2]. Although surgery, chemotherapy and radiotherapy for the treatment of CRC have substantially improved in recent decades, the overall survival rate and 5-year survival rate of patients remain poor [3]. Invasion or metastasis to distant organs, such as the lung and liver, is common and is the main cause of death in patients with CRC [4, 5]. The initiation and progression of CRC are governed by a complex network that involves multiple molecules and pathways. The classic adenoma-carcinoma model of CRC is initiated by the progressive accumulation of genetic alterations, which subsequently result in the malignant transformation of normal colorectal epithelial cells into adenocarcinoma cells [6]. Nevertheless, further research is required to identify the underlying mechanisms involved in the development and progression of CRC.

MicroRNAs (miRNAs) are small noncoding RNAs that usually negatively regulate gene expression by degrading target mRNAs, inhibiting the translation of these mRNAs, or both [7]. Many studies have demonstrated that miRNAs can function as oncogenes or tumor suppressors and are often dysregulated in tumors [8]. miRNAs play a vital role in many crucial cellular processes, including apoptosis, differentiation, invasion and proliferation [9–11]. The abnormal expression of miRNA has been reported in many cancers, including breast cancer [12], gastric cancer [13], colorectal cancer [14], liver cancer [15], leukemia [16], and lymphoma [17].

miR-124 and miR-506 have also been reported to function as important regulators in many human cancers [18–20]. Specifically, both miR-124 [21] and miR-506 [22] are reportedly involved in the epithelial-mesenchymal transition (EMT), which is considered to influence and promote certain cell motility and metastasis steps during tumor progression [23, 24]. However, the pathological correlations and biological functions of miR-124 and miR-506 in the control of colorectal tumourigenesis have not been characterized.

DNA methylation is a postreplicative modification that usually occurs in prokaryotic and eukaryotic genomes. It has been reported to be involved in a variety of vital biological functions, such as the regulation of gene expression, inactivation of the X-chromosome, and preservation of chromosomal integrity [25–28]. DNA methylation is the enzymatic addition of a methyl group to the carbon at the 5 position of cytosine in the context of the sequence 5′-cytosine-guanosine (CpG), and this process is mediated by DNA methyltransferases (DNMTs: DNMT3A, DNMT3B and DNMT1) [29, 30]. The promoter regions of approximately 50% of human genes contain DNA regions with greater cytosine and guanine contents than expected. The hypermethylation of these regions mediates gene transcriptional silencing [30, 31]. The silencing of structurally normal tumor suppressor genes by aberrant DNA hypermethylation has been reported in cancer therapy, such as those used to treat gastric cancer [32]. Furthermore, the association of miRNAs with DNA methylation has been reported in many human cancers. Specifically, miR-152 inhibits liver fibrosis by attenuating DNMT1-mediated Patched1 methylation [33]. Hepatocarcinogenesis is also reportedly regulated by DNA methylation via microRNAs [34]. miR-124 has also been reported to target SP1, which is associated with DNA methylation [35].

An increasing number of studies have revealed that global DNA hypomethylation and regional hypermethylation frequently occurs during tumorigenesis [36–38], which indicates that aberrant methylation substantially contributes to cancer initiation or progression.

In this study, we found that miR-124 and miR-506 were strongly down-regulated in CRC tissues and cell lines. Functional studies identified miR-124 and miR-506 acted as new tumor suppressors in CRC. Moreover, we found that miR-124 and miR-506 targeted DNMT3B and DNMT1 (SP1 is a transactivator of the DNMT1 gene), which markedly reduced the expression of DNMT3B, DNMT1 and SP1 at both the RNA and protein levels. In turn, this reduced expression led to a decrease in global DNA methylation and the re-expression of the tumor suppressor genes E-cadherin, MGMT (O-6-methylguanine-DNA methyltransferase) and P16 via promoter DNA hypomethylation.

## RESULTS

### miR-124 and miR-506 are downregulated in colorectal cancer

**Figure 1:**
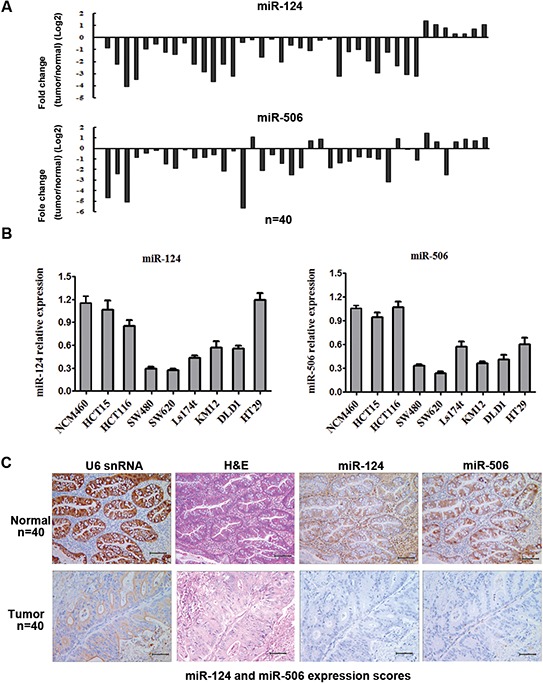
MiR-124 and miR-506 expression levels are frequently downregulated in human CRC **A.** Expression levels of miR-124 (top) and miR-506 (bottom) in 40 CRC patients were detected by qRT-PCR. Data are shown as the log2 of fold change of CRC relative to adjacent normal tissues. **B.** Relative expression of miR-124 (left) and miR-506 (right) in 8 cell lines derived from CRC and a cell line derived from normal colonic epithelium was determined by qRT-PCR. Data are presented as the mean ± SD from at least three separate experiments. **C.** Representative images of miR-124 and miR-506 expression by ISH. Scale bars: 100 μm.

The expression levels of miR-124 and miR-506 were detected by qRT-PCR in 40 pairs of CRC tissues and their matched adjacent tissues, as well as in CRC cell lines. Among samples from 40 patients with CRC, approximately 82.5% (*P* = 0.000, 33 of 40 patients) and 75% (*p* = 0.000, 30 of 40 patients) of tumor tissues revealed notable reductions in the miR-124 and miR-506 levels, respectively (Figure 1A). The miR-124 and miR-506 expression levels in eight CRC cell lines were measured by qRT-PCR. The results showed that the expression levels of miR-124 and miR-506 were significantly lower in SW620 and SW480 cells than in NCM460 normal colonic epithelium cells (Figure 1B). Therefore, we over-expressed miR-124 and miR-506 in SW620 and SW480 cells to evaluate the possible role of miR-124 and miR-506 in CRC pathogenesis. To further verify the biological roles of miR-124 and miR-506 in human CRC, we performed *in situ* hybridization (Figure 1C) to evaluate the miR-124 and miR-506 levels in 40 CRC tissues and 40 normal colon tissues and found that miR-124 and miR-506 were strongly downregulated in CRC tissues compared with normal tissues. Among samples from 40 patients with CRC, approximately 65% (*P* = 0.000, 26 of 40 patients) and 70% (*P* = 0.000, 28 of 40 patients) of tumors revealed notable reductions in the miR-124 and miR-506 levels, respectively. These data indicate an overt downregulation of miR-124 and miR-506 in CRC.

### Overexpression of miR-124 or miR-506 inhibits tumor cell progression and increases sensitivity to chemotherapy *in vitro*

**Figure 2:**
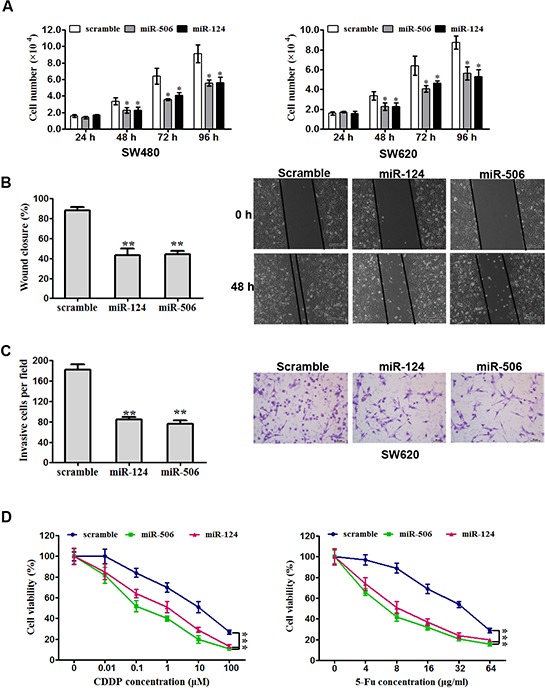
Overexpression of miR-124 or miR-50b inhibits tumor cell progression and increases sensitivity to chemotherapeutics *in vitro* **A.** SW620 (left) and SW480 (right) cells were transfected with miR-124 mimic, miR-506 mimic or scrambled control. They were then seeded in 12-well plates at a desired cell concentration and maintained in medium containing 10% fetal bovine serum. The cells were counted in triplicate at the indicated time points, and their growth rates were recorded. All data are presented as the mean ± s.e.m., *P < 0.05. **B.** Scratch wound assays were performed on SW620 cells transfected with miR-124 mimic, miR-506 mimic or scrambled control. The data from three separate assays were averaged and then graphed. All data are presented as the mean ± s.e.m., **P < 0.01. **C.** An invasion assay was used to quantify cell invasion in a Matrigel-coated chamber. The average number of migrated per field of view from three different experiments is plotted, as described in Materials and methods. Scale bar: 200 mm. All data are presented as the mean ± s.e.m., **P < 0.01. **D.** An MTT assay was used to evaluate cell viability. Forty-eight hours after transfection with miR-124 mimic, miR-506 mimic or scrambled control, SW620 cells were exposed to a range of CDDP or 5-FU concentrations for 24 h, and the cell viability was determined and recorded. Data are presented as the mean ± SD from at least three separate experiments. ***P < 0.001.

The ectopic expression of miR-124 or miR-506 has been reported to be associated with the progression in many tumors [18–24]. To assess the biological effects of overexpressing miR-124 or miR-506 in CRC cells, miR-124 or miR-506 mimic was transfected into SW620 and SW480 cells. The transfections of miR-506 and miR-124 were successful (Supplement Figure S1). Compared with the scrambled control transfection, the overexpression of either miR-124 or miR-506 significantly attenuated the proliferation of the two cell lines (Figure 2A). Furthermore, the overexpression of miR-124 or miR-506 markedly inhibited the ability of SW620 cells to migrate (Figure 2B), and the overexpression of either miR-124 or miR-506 remarkably attenuated cell invasion in SW620 cells (Figure 2C, five fields were counted in the Matrigel-coated cell invasion experiments). Cisplatin (CDDP) and 5-fluorouracil (5-FU) are usually used to treat CRC [39]; thus, we measured cell viability to assess the response to these agents. Specifically, we measured the IC50 values using MTT assays. The IC50 values of CDDP and 5-FU in SW480 cells were 2 × 10^−7^ μg/mL and 32 μg/mL, respectively. We found that the overexpression of miR-124 or miR-506 increased the sensitivity of CRC cells to these two agents (Figure 2D). The effects of miR-124 and miR-506 inhibitors on tumor cell proliferation were also tested. We found that miR-124 and miR-506 inhibitors decreased the sensitivity of CRC cells to these two agents (Supplementary Figure S1 and S2).

### Overexpression of miR-124 or miR-506 inhibits tumor cell proliferation and invasion *in vivo*

**Figure 3:**
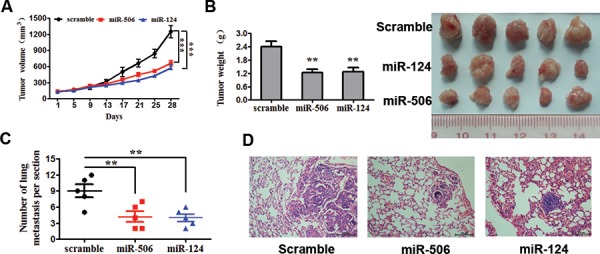
Overexpression of miR-124 or miR-506 inhibits tumor cell proliferation and invasion *in vivo* **A.** SW620 cells infected with miR-124, miR-506 or scramble control were subcutaneously injected into the flanks of nude mice (*n* = 5 for each group). After injection, the average tumor volume was measured every four days until the mice were killed 28 days later. The growth curves of the tumors were then plotted. All data are presented as the mean ± s.e.m., ***P < 0.001. **B.** After 28 days, all mice were euthanized to excise the xenograft. Necropsies were performed, and the tumors were weighed. All data are presented as the mean ± s.e.m., *P < 0.05. **C.** Number of lung metastases per section. The number of metastatic nodules in five mice in each team was counted under the microscope. *P < 0.05. **D.** Representative images of lung metastasis of the miR-124-overexpressing, miR-506-overexpressing and scrambled control groups. Scale bars: 100 μm.

The above results prompted us to verify that miR-124 or miR-506 inhibit CRC tumor cell proliferation and invasion *in vivo*. To this end, a xenograft model of CRC cells in nude mice was constructed. SW620 cells infected with miR-124, miR-506 or scrambled control were subcutaneously injected into the flanks of nude mice (five in each group). The tumor volume was measured every four days after injection. The growth curves of the tumors were plotted accordingly the recorded data. After 28 days, all mice were euthanized to excise the xenograft. The mean volume and weight of tumors were significantly lower in the miR-124- and miR-506-overexpressing groups than in the control group (Figure 3A, 3B). Necropsies were performed, and the number of metastatic nodules in each mouse was counted under a microscope. The ectopic expression of miR-124 or miR-506 dramatically decreased the number of lung metastases in mice (Figure 3C). Representative images of lung metastases for the miR-124-overexpressing, miR-506-overexpressing and control groups are shown in Figure 3D. The above results suggest that miR-124 or miR-506 inhibit CRC tumor cell proliferation and invasion *in vivo*.

### miR-124 and miR-506 directly target DNMT3B and indirectly target DNMT1

**Figure 4:**
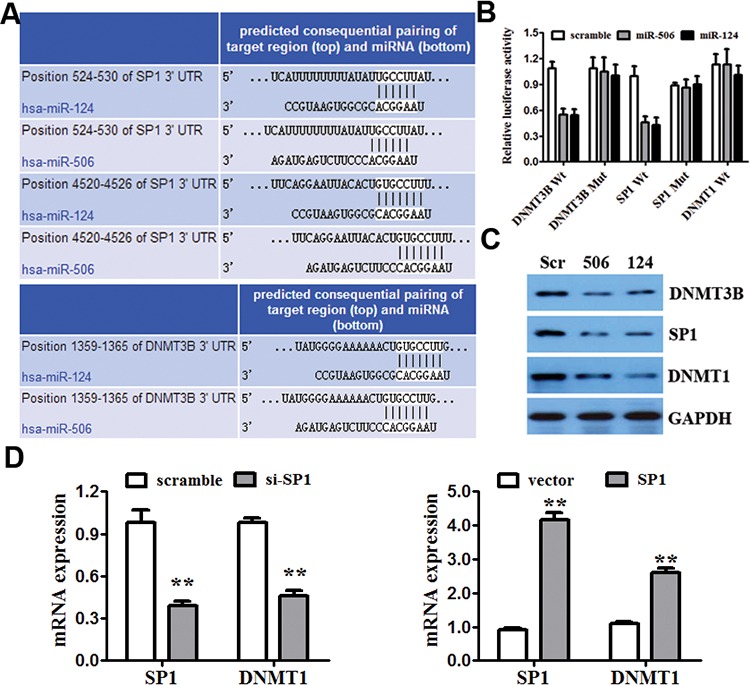
miR-124 and miR-506 directly target DNMT3B and indirectly target DNMT1 **A.** The 3′UTRs of DNMT3B and SP1 contain putative binding sites for miR-124 and miR-506. **B.** A luciferase assay of SW620 cells that were co-transfected with miR-124 mimic, miR-506 mimic or scrambled control and a luciferase reporter containing the following: DNMT3B 3′-UTR (DNMT3B-wt), SP1 3′-UTR (SP1-wt), DNMT1 3′-UTR (DNMT1-wt) or mutant constructs in which the first four nucleotides of the miR-124 and miR-506 binding site were mutated: DNMT3B-mut and SP1-mut. An empty luciferase reporter construct was used as a negative control. *P < 0.05 vs. scrambled control. **C.** The effect of miR-124 and miR-506 on the protein expression of DNMT3B, SP1 and DNMT1 by western blot. GAPDH was used as a loading control. **D.** The effect of the SP1 siRNA and overexpression SP1 on the mRNA expression of DNMT1 and SP1 by qRT-PCR in SW620 cells. GAPDH was used as a control. *P < 0.05, **P < 0.01.

To understand the mechanism by which miR-124 and miR-506 suppress CRC growth and invasion, we used two algorithms (Targetscan and Miranda) to help identify miR-124 and miR-506 targets in CRC. Both algorithms predicted DNMT3B and SP1 as target genes (Figure 4A). SP1 has been reported to regulate the transcription and expression of DNMT1 and is associated with the cell cycle, proliferation and invasion [40, 41]. We hypothesized that miR-124 and miR-506 may directly target DNMT3B and may indirectly target DNMT1. We confirmed this hypothesis in CRC cells by performing luciferase reporter assays. The DNMT3B, SP1 and DNMT1 complementary sites were cloned downstream of the firefly luciferase gene and cotransfected with miR-124 mimic, miR-506 mimic or scrambled oligonucleotide. The luciferase activity was then measured 48 h after transfection. Transfection with miR-124 and miR-506, compared with the scrambled oligonucleotide, distinctly reduced the luciferase activity in SW620 cells that were co-transfected with either DNMT3B or SP1 but not DNMT1 reporter constructs (Figure 4B). Moreover, mutating the putative miR-124 and miR-506 sites in the 3′-UTR of DNMT3B and SP1 abrogated the luciferase responsiveness to miR-124 and miR-506 (Figure 4B). We also found that transfecting the miR-124 and miR-506 mimics into SW620 cells markedly decreased the protein levels of DNMT3B and SP1 (Figure 4C). In contrast to DNMT3B and SP1, miR-124 and miR-506 are not predicted to hybridize with the DNMT1 3′UTR region. However, transfecting miR-124 and miR-506 into SW620 cells also generated a marked decrease in DNMT1 protein levels (Figure 4C). The above results may preliminarily indicate that DNMT3B is a direct target and that DNMT1 is an indirect target of miR-124 and miR-506. SP1 is a zinc finger transcription factor that directly binds to the promoter of DNMT1. SP1 has been reported to positively regulate the transcription of DNMT1 in mice [42]. We further confirmed the link between DNMT1 and SP1 by showing that SP1-siRNA and the overexpression of SP1 in SW620 cells resulted in the downregulation of SP1 and the subsequent downregulation of the DNMT1 mRNA levels, as measured by qRT-PCR (Figure 4D).

### Overexpression of miR-124 and miR-506 reduces global DNA methylation and restores the expression of E-cadherin, MGMT and P16

**Figure 5:**
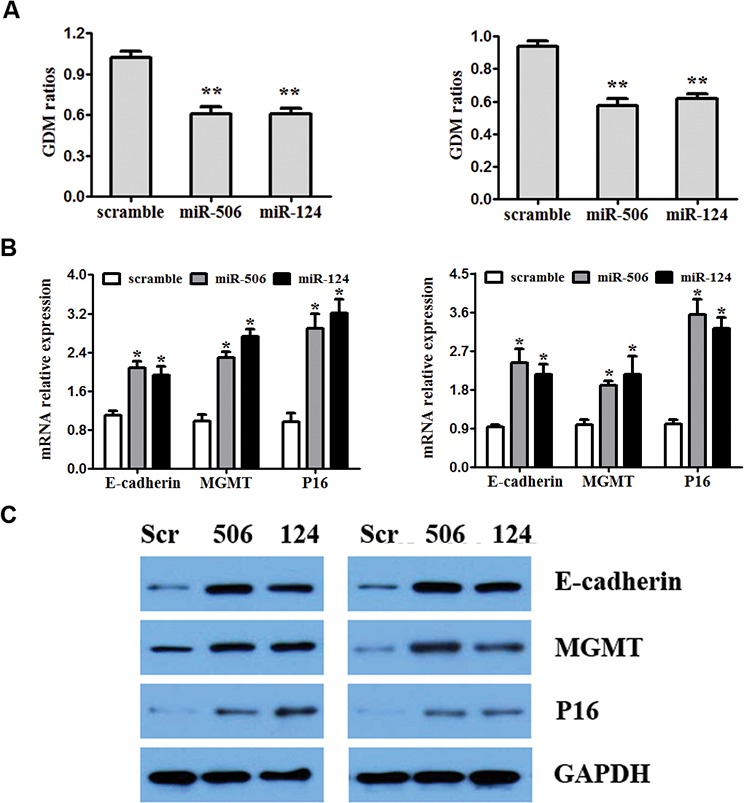
Overexpression of miR-124 and miR-506 reduces global DNA methylation and restores the expression of E-cadherin, MGMT and P16 **A.** SW480 (left) and SW680 (right) cell were transfected with miR-124 mimic, miR-506 mimic or scrambled control. Forty-eight hours after the transfection, DNA was obtained from the cells, and absolute GDM was measured by HPLC-DAD. Bars represent the range of 3 independent experiments. **B.** SW480 (left) and SW680 (right) cells were transfected with miR-124 mimic, miR-506 mimic or scrambled control. The mRNA expression of E-cadherin, MGMT and P16 was detected by qRT-PCR. **C.** The effect of the miR-124 or miR-506 mimics on the protein expression of E-cadherin, MGMT and P16, determined by western blotting in SW480 and SW680 cells. GAPDH was used as a loading control.

**Figure 6:**
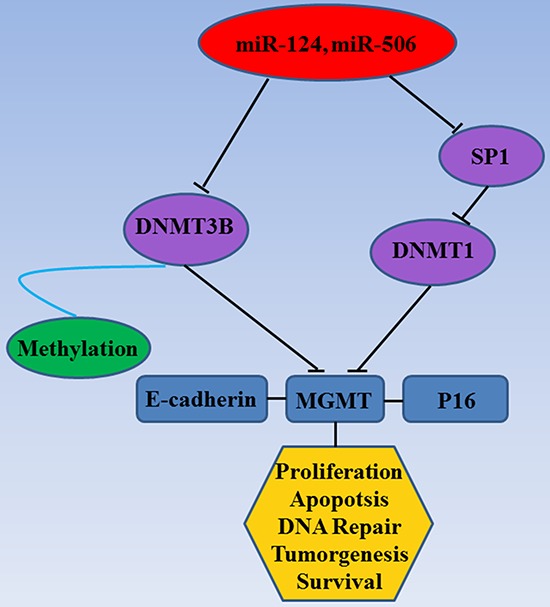
Mechanism diagram Diagram of the mechanism by which miR-124 and miR-506 inhibit progression by targeting DNMT3B and DNMT1 in CRC.

To investigate the effect of miR-124 and miR-506 overexpression on DNA hypomethylation, we measured global DNA methylation (GDM) in SW480 and SW620 cells by HPLC-DAD [43] 48 hours after transfection with miR-124 mimic, miR-506 mimic and the scrambled control. A significant reduction was observed in the GDM when SW480 and SW620 cells were treated with miR-124 and miR-506 mimics compared with the controls (Figure 5A). To assess whether overexpression of miR-124 or miR-506 leads to the re-expression of hypermethylated and silenced genes in CRC, we measured the mRNA and protein levels of E-cadherin, MGMT and P16 in SW480 and SW420 cells by qRT-PCR and western blotting after transfection with miR-124, miR-506 or scrambled control. Specifically, the E-cadherin, MGMT and P16 mRNA levels were increased compared with the control (Figure 5B), and these changes persisted at the protein level (Figure 5C). A diagram of the mechanism by which miR-124 and miR-506 inhibit progression by targeting DNMT3B and DNMT1 in CRC is shown in Figure 6.

## DISCUSSION

Several reports have indicated that either miR-124 or miR-506 plays a significant role in growth, metastasis and proliferation [18–20]. Specifically, miR-124 expression is reportedly downregulated in the cells and tissues of esophageal cancer [44] breast cancer [45] and renal cell carcinoma [46]. Furthermore, miR-506 expression is reportedly downregulated in oral squamous cell carcinoma [47], nasopharyngeal carcinoma [48] and gastric cancer [49]. However, miR-124 and miR-506 have rarely been studied in the context of CRC. In this study, we showed that the miR-124 and miR-506 levels were significantly lower in CRC tissues than in normal tissues, as indicated by qRT-PCR and *in situ* hybridization histochemistry (ISH). These results suggested that the abnormal expression of miR-124 and miR-506 are associated with CRC.

Furthermore, we studied the biological effects of the overexpression of miR-124 and miR-506 in CRC. The *in vitro* data demonstrated that miR-124 and miR-506 function as tumor suppressors in CRC. Specifically, the overexpression of miR-124 and miR-506 inhibits colorectal tumor cell proliferation, migration, and invasion while also increasing drug sensitivity *in vitro*. These findings corroborate results from many previous studies. For example, miR-124 has been shown to inhibit cell proliferation and suppress tumor growth in breast cancer [50], and miR-506 has been shown to tumor proliferation and invasion in nasopharyngeal carcinoma [47]. This inhibition was also verified in a xenograft model of CRC in nude mice *in vivo*. Therefore, restoring miR-124 and miR-506 expression in CRC inhibits tumor progression *in vitro* and *in vivo*.

Moreover, the roles of miR-124 and miR-506 in the regulation of DNA methylation in CRC were characterized. Our data showed that miR-124 and miR-506 target DNMTs (DNMT3B and DNMT1), thus leading to global DNA hypomethylation in CRC. SP1 regulates a large number of genes associated with the cell cycle, proliferation and invasion [40, 41]. SP1 can bind to the promoter of DNMT1 and transactivate the DNMT1 gene in mice [42]. Our research demonstrated that miR-124 and miR-506 directly down-regulate DNMT3B and indirectly down-regulate DNMT1 by targeting SP1; that is, miR-124 and miR-506 directly target DNMT3B and indirectly target DNMT1.

The down-regulation of DNMT3B and DNMT1 by miR-124 and miR-506 has important functional ramifications. Selective genetic disruption of DNMT3B has been shown to reduce GDM by only 3%, but the genetic disruption of both DNMT1 and DNMT3B has been shown to reduce GDM by 95% and completely abrogate DNA methyltransferase activity [30, 51]. Our study showed that miR-124 and miR-506 efficiently modulate DNA hypomethylation by targeting DNMT3B and DNMT1. Furthermore, the overexpression of miR-124 or miR-506 results in global DNA hypomethylation and gene re-expression of the hypermethylated and silenced E-cadherin, MGMT and P16 genes in CRC.

In conclusion, miR-124 and miR-506 may be valuable markers of CRC prognosis and may play an important role in the development and progression of human CRC.

## MATERIALS AND METHODS

### Cell culture

The human CRC cell lines SW480, DLD1, HCT116, HCT15, KM12 and Ls174t were obtained from the Chinese Academy Medical Science (Beijing, China) and maintained at 37°C in an atmosphere of 5% CO_2_ in RPMI-1640 medium (Gibco, USA) containing 10% fetal bovine serum (FBS; PAA Laboratories, Austria); SW620 and HT29 cells were cultured in Dulbecco's modified Eagle's medium (DMEM; Gibco, USA) supplemented with 10% FBS.

### Clinical samples

Prior approval was obtained from The Institutional Review Board of Third Xiangya Hospital, Central South University (Changsha, Hunan, China) for the collection and use of clinical materials for research purposes. All tissue samples used in this study were collected from the Third Xiangya Hospital (Changsha, Hunan, China). Written informed consent was obtained from all study participants. Forty CRC tissues and the matched adjacent normal tissues were obtained between 2012 and 2013. All tissue biopsies used here were freshly frozen in liquid nitrogen and stored at −80°C until further use.

### RNA extraction and qRT-PCR

The total RNAs from cells and tissues were extracted with the mirVana miRNA Isolation Kit (Ambion, USA) according to the manufacturer's protocol. The cDNA was synthesized from total RNA using a TaqMan miRNA Reverse transcription kit (Applied Biosystems, USA). qRT-PCR was performed using iQ^TM^ SYBR Green Supermix (Bio-Rad, USA) with 5 ng cDNA and 10 pM of each primer and detected on an Applied Biosystems 7500 Sequence Detection system. The following cycling conditions were used: one cycle at 94°C for 5 min; 40 cycles of 95°C for 30 s and 56°C for 30 s. A melting curve analysis was carried out for each PCR reaction to confirm the specificity of amplification. The expression of miRNA was calculated based on the threshold cycle (CT), and after normalization to level of U6 small nuclear RNA expression, the relative expression levels were calculated as follows: 2^−[(CT ofmi^R-30b)−(CT ofU6)]. The U6 snRNA was 5′-ATTGGAACGATACAGAGAAGATT-3′. qRT-PCR for the target genes was performed as previously described [52].

### ISH analysis

ISH procedures were carried out as previously described [13]. Briefly, miR-124 and miR-506 miRCURY LNA custom detection probes (Exiqon, Denmark) were used for ISH. Hybridization, washing and scanning were carried out according to the manufacturer's instructions. The intensities of miR-124 and miR-506 staining were scored on a scale from 0 to 4 as follows: 0–1 (no staining), 1–2 (weak staining), 2–3 (medium staining), and 3–4 (strong staining). The percentages of miR-124 and miR-506 cells in 3 representative high-power fields of individual samples were analyzed. The expression scores were determined by multiplying the scores of the intensities by the percentages of positive cells and ranged from a maximum of 4 to a minimum of 0. Individual samples were evaluated by at least 2 pathologists in a blinded manner, and expression scores greater or equal to 2 were defined as high expression, whereas scores lower than 2 were indicative of low expression.

### Plasmids and transfection

To generate the miR-124 and miR-506 expression vectors, a genomic fragment covering the region encoding pri-miR-124 or pri-miR-506 and its up-and downstream region were PCR-amplified and cloned into the pLvthm vector (Addgene Inc, USA). The vectors were generated by PCR amplification and subcloned into the Bam HI/Sal I sites of the pGL3–basic luciferase reporter plasmid (Promega, USA). miR-124 mimic, miR-506 mimic and scrambled oligonucleotides were purchased from Genecopoeia (China) and transfected into CRC cells using Lipofectamine 2000 reagent (Invitrogen, USA) according to the manufacturer's instructions. Two concentrations of miR-124 mimic and miR-506 mimic (10 and 50 nM) were tested.

### SP1 silencing

The following sense sequence of siRNA oligonucleotides was used to target the SP1 transcripts: si-SP1: 5′-CACAAACACTGCCCACCG-3′ (Invitrogen, USA). Scrambled siRNA was used as a negative control. Cells were plated in culture dishes for 24 h and transfected with siRNA using Lipofectamine 2000. After 48 h, the cells were harvested for use in other assays or for RNA and protein extraction.

### Western blot

Protein was extracted from CRC cell lines using RIPA lysis buffer containing a proteinase inhibitor. The protein concentration in the lysates was measured with the Protein BCA Assay Kit (Bio-Rad, USA), and 20 μg of protein mixed with 2 × SDS loading buffer was loaded in each lane. The proteins in the lysates were then separated by 12% SDS-polyacrylamide gel electrophoresis and transferred to polyvinylidene difluoride membranes (Millipore, USA). To block nonspecific binding, membranes were incubated at room temperature for 1 h in 5% skim milk. Next, the membranes were incubated for 12 h at 4°C with an antiserum containing antibodies against DNMT1, DNMT3A, DNMT3B, SP1, E-cadherin, MGMT or P16, which were purchased from Santa Cruz Biotechnology (Santa Cruz, USA). A peroxidase-conjugated secondary antibody (1:5000 dilution) and ECL western blotting detection reagents were used to visualize the target proteins (ECL New England Biolabs, USA), and the resultant signals were quantified with a Bio Image Intelligent Quantifier 1-D (Version 2.2.1, Nihon-BioImage Ltd., Japan). An anti-GAPDH antibody (Boster, China) was used as a protein loading control.

### Cell proliferation assay

Cells transfected with miR-124 mimic, miR-506 mimic and scrambled oligonucleotides (Ambion, USA) were plated in 12-well plates at the desired cell concentrations. Cell counts were estimated by trypsinizing the cells and counting them in triplicate using a Coulter Counter (Beckman Coulter, USA) at the indicated time points.

### Cell migration assay

Cell migration was examined by wound-healing assays. An artificial “wound” was created by scratching a confluent cell monolayer of cells, and the scratched cells were then treated with 10 mg/mL mitomycin C for 2 hours. Photographs were taken after 24 hours using an inverted microscope (Olympus, Japan).

### Cell invasion assay

The cell invasion assay was conducted as previously described [53]. Cells were seeded onto the basement membrane matrix present in the insert of a 24-well culture plate (EC matrix; Chemicon, Japan). After an additional 48 hours, the noninvading cells and EC matrix were gently removed with a cotton swab. Invasive cells located on the lower side of the chamber were stained with crystal violet, counted, and imaged.

### GDM analysis

GDM was analyzed as previously described [43]. Briefly, genomic DNA was isolated from SW480 and SW620 CRC cells using a genomic DNA extraction kit according to the manufacturer's instructions (TaKaRa, Japan). High-performance liquid chromatography/diode array detectors (HPLC-DAD) were used to determine the levels of GDM in each sample.

### Xenograft model in nude mice

SW620 cells were used to stably over-express miR-124 and miR-506 using a lentiviral-based system (pLVTHM) for tumourigenesis assays. Xenograft tumors were generated via the subcutaneous injection of CRC cells (2 × 10^6^, SW620/scramble or SW620/miR-506) into the hind limbs of 4–6 week-old Balb/C athymic nude mice. All mice were housed and maintained under specific pathogen-free conditions, and all experiments were approved by the Use Committee for Animal Care and performed in accordance with institutional guidelines. Tumor size was measured by a slide caliper, and tumor volume was determined using the formula: 0.44 × A × B^2^ (A is the diameter of the base of the tumor, B is the corresponding perpendicular value). The tumors were excised after euthanasia, fixed in 10% neutral buffered formalin, and embedded in paraffin before preparing 4 μm sections, which were stained with hematoxylin.

### Statistical analysis

All statistical analyses were performed using SPSS19.0. The two-tailed paired Student's *t*-test was used to analyze the two groups. The Mann–Whitney *U*-test and Spearman's correlation analyses were used to analyze the relationship between miR-124 and miR-506 expression and the clinicopathological features of CRC. Survival curves were plotted by the Kaplan–Meier method and compared with the log-rank test. *P* < 0.05 was considered to indicate significant differences.

## SUPPLEMENTARY FIGURES


