# Supplementary material for: miR-124 and miR-506 inhibit colorectal cancer progression by targeting DNMT3B and DNMT1
Source: Oncotarget. 2015 Oct 15;6(35):38139–50. doi: 10.18632/oncotarget.5709 (PMC4741989; doi:10.18632/oncotarget.5709)
Supplement: Supplementary file 1 [file oncotarget-06-38139-s001.pdf]

SUPPLEMENTARY FIGURES

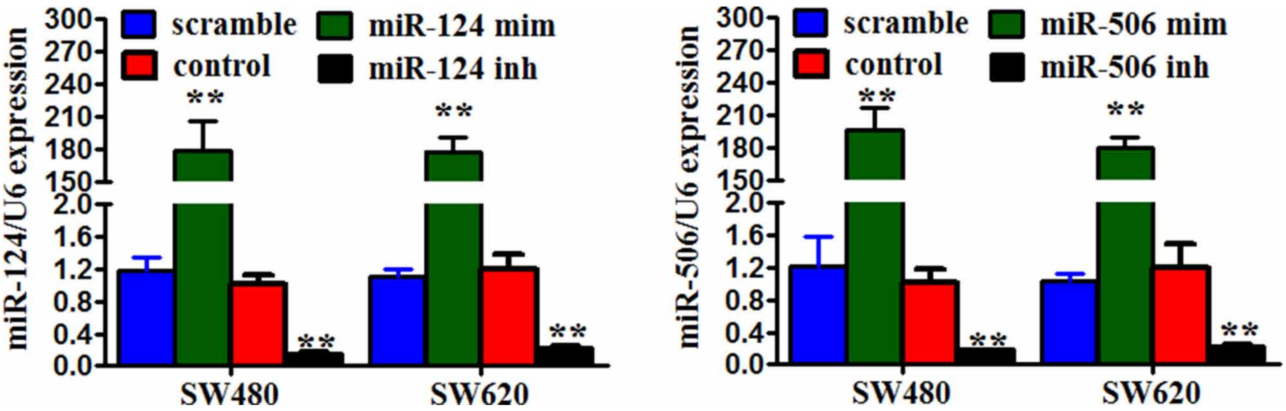

Supplementary Figure S1: The transfection was successful. The transfections of miR-506 mimic, miR-124 mimic, miR-506 inhibitor and miR-124 inhibitor were successful. All data are presented as the mean ± s.e.m., $^{**}P < 0.01$.

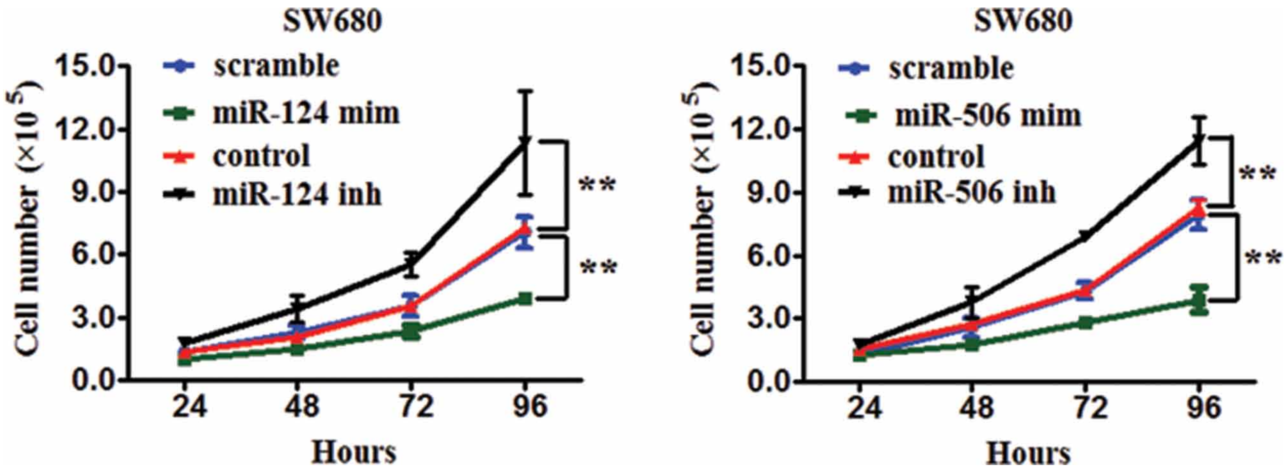

Supplementary Figure S2: The effects of miR-124 and miR-506 inhibitors on tumor cell progression. We found that the miR-124 and miR-506 inhibitors decreased the sensitivity of CRC cells to the two agents. All data are presented as the mean ± s.e.m., $^{**}P < 0.01$.
